# 임부의 e헬스 문해력, 인터넷 건강정보에 대한 태도가 공유의사결정에 미치는 영향: 단면연구

**DOI:** 10.4069/whn.2025.06.19

**Published:** 2025-06-30

**Authors:** Minju Kim, Gungeong Lee

**Affiliations:** College of Nursing, Ewha Womans University, Seoul, Korea; 이화여자대학교 간호대학

**Keywords:** Attitude to health, Decision making, Health literacy, Pregnancy, 건강문해력, 건강태도, 의사결정, 임부

## Abstract

**Purpose:**

This study investigated eHealth literacy and attitudes toward internet health information among pregnant women and analyzed the associations between these factors and shared decision-making related to pregnancy and birth.

**Methods:**

The participants were 130 pregnant women receiving care at an obstetrics and gynecology department of a tertiary general hospital in Seoul, Korea. Data on eHealth literacy, attitudes toward internet health information, and shared decision-making were collected via an online survey conducted in October 2024. The collected data were analyzed using frequency analysis, descriptive statistics, Pearson correlation coefficients, the independent t-test, one-way analysis of variance, and hierarchical multiple regression.

**Results:**

The mean age of participants was 31.73±2.86 years. Thirty percent of the participants were in early pregnancy, 37.7% in mid-pregnancy, and 32.3% in late pregnancy. The mean eHealth literacy score was 4.11±0.75, indicating a high level. The average score for attitudes toward internet health information was 4.10±0.73, reflecting positive attitudes. The mean score for shared decision-making was also high, at 4.17±0.73. Higher eHealth literacy and more positive attitudes toward internet health information were found to significantly influence shared decision-making (F=41.03, *p*<.001), explaining 46.3% of the variance.

**Conclusion:**

A high level of eHealth literacy and positive attitudes toward internet health information are important for promoting shared decision-making among pregnant women. Public institutions should offer educational programs that improve the ability to search for and utilize health information. In clinical settings, specialized counselors should be employed to provide personalized education and accurate information supporting informed maternal health decisions.

## Introduction

건강한 아이의 출산은 산모와 신생아의 건강은 물론 사회 전체의 건강증진을 위한 중요한 기초가 되며, 특히 저출산 문제 해결에도 핵심적인 역할을 한다[1]. 우리나라의 경우 평균 초혼 연령이 높아짐에 따라 고령 출산율이 증가하고 있으며[2], 고령 임부는 임신 중 당뇨, 고혈압, 전치태반 등의 임신 합병증 발생 위험이 크고, 신생아에게 조산, 사산, 영아 사망 등의 영향을 미칠 수 있다[3]. 따라서 임부들은 임신 기간 동안 경험하는 신체적 변화와 생리적 요인이 정상적인지 확인하기 위해 적극적으로 정보를 찾게 된다[4]. 과거에는 가족이나 지인들, 의료기관, 서적, 대중매체를 통해 정보를 얻었으나[5] 최근에는 인터넷을 통해 쉽고 빠르게 다양한 정보를 얻는 경향이 강하다[6]. 또한 팬데믹을 겪으면서 의료기관 이용이 감소함에 따라 임부들의 인터넷을 통한 건강정보 검색은 3배 이상 증가한 것으로 나타났다[7].

임신 중에 발생하는 증상이나 불확실한 상황은 임부들에게 심리적인 불안을 초래할 수 있다[8]. 이들은 불안을 해소하고 필요한 정보를 얻기 위해 의료진과 상담 대신 인터넷에서 비슷한 경험을 공유한 여성들의 이야기를 참고하거나 지식을 습득하며 자신의 상태를 예측하려 한다[9]. 그러나 이러한 정보는 의료 서비스 제공자와 논의 없이 임부들이 자체적으로 판단하고 수용하는 경우가 많고[10], 전문가와 상의 없이 인터넷 정보를 따를 경우 건강에 위험을 초래할 수 있으며[11], 과도한 정보에 노출되면 오히려 건강정보에 대한 신뢰가 떨어지거나 혼동을 일으켜 불안감이 증가될 수 있다[12].

e헬스 문해력은 인터넷을 통해 적절한 건강정보를 찾고 평가하며 이를 이해하고 활용하는 능력을 의미한다[13]. 선행연구에 의하면 여성인 경우, 교육 수준이 낮은 경우, 그리고 e헬스 문해력이 낮은 경우 건강정보를 부정확하게 해석할 가능성이 높았다[14]. 그러나 e헬스 문해력이 높은 사람은 모바일이나 인터넷을 통해 건강 관련 질문에 대한 답을 찾고 그 정보를 사용하여 건강행동을 개선하는 데 더 관심을 가지는 것으로 나타났다[15]. 인터넷을 통한 건강정보 수집은 시간과 장소에 구애받지 않고 빠르게 접근할 수 있다는 장점이 있지만, 잘못된 정보에 노출될 가능성도 크다[14]. 정보 검색 시 적절한 정보원을 활용하고 정보를 바르게 해석 및 적용할 수 있는 능력은 건강과 밀접한 관련이 있으며, 이를 위해서는 e헬스 문해력이 필수적이다[7].

인터넷은 현대 사회에서 건강정보를 얻는 중요한 도구로 자리 잡았으며, 특히 임신∙출산과 같은 민감한 건강 문제에 관심이 높은 집단에서는 적극적으로 활용되고 있다[16]. 선행연구에 따르면 긍정적인 인터넷 건강정보에 대한 태도는 사람들이 건강 행위에 참여하도록 자극할 수 있으며[12,17], 건강증진 의지가 강할수록 질병 관련 정보를 인터넷에서 더욱 적극적으로 활용하는 경향이 있다[12]. 건강에 관한 관심이 높을수록 사람들은 인터넷 건강정보를 더 유용하다고 생각하고, 진료 과정이나 건강 예방 활동을 결정하는 데에도 이를 활용할 가능성이 크다[18]. 그러나 인터넷에서 제공되는 건강정보는 출처와 신뢰성이 다양하여 부정확하거나 왜곡된 정보가 포함될 수 있는 위험이 존재하며[10], 이러한 정보는 임부들의 건강관리 및 의사결정에 혼란을 초래할 수 있다[19]. 이에 임부들의 인터넷 건강정보에 대한 태도를 조사하고 이해하는 것은 매우 중요하다고 볼 수 있다.

현대 의료 환경에서는 환자의 권리와 자기결정권에 대한 인식이 확대되면서, 단순한 수동적 진료 수혜자가 아닌, 의료 의사결정에 적극적으로 참여하는 주체로서의 환자의 역할이 강조되고 있다[20]. 이러한 흐름 속에서 등장한 공유의사결정은 환자가 스스로 수집한 정보를 바탕으로 의료진과 상호작용하며 진료 및 치료 방침에 대해 함께 결정하는 과정으로 정의되며, 이는 환자 만족도와 치료 수행률을 높이는 효과가 있는 것으로 평가된다[21].

인터넷의 발달은 환자가 의료정보를 보다 쉽게 수집할 수 있도록 하여, 공유의사결정의 가능성과 효과를 더욱 확대하는 요인이 되었다[22]. 공유의사결정은 의료진과 환자가 단순히 정보를 공유하는 것을 넘어 가치와 선호를 반영한 정보 교환을 통해 환자가 자기결정권을 행사하도록 돕는 윤리적•합리적 의료 의사결정 방식으로 자리매김하고 있다[20,23]. 그러나 인터넷에는 검증되지 않은 정보 또한 범람하고 있어, 환자가 이러한 정보에 의존하거나 집착할 경우 의료진의 전문적 판단을 불신하거나 의료적 판단 과정에 혼란을 초래할 수 있다는 지적도 있다[24,25]. 반면 인터넷 건강정보에 대한 비판적 수용 능력과 정보 이해도가 높은 환자들은 의료진과의 상호작용에서 더욱 적극적인 의사결정을 수행하며, 자신의 건강관리와 치료에 주도적으로 참여하는 모습을 보였다[26,27]. 이러한 경향은 환자들이 단순히 정보를 소비하는 데 그치지 않고, 공유의사결정의 중심 주체로 기능할 수 있음을 시사한다. 실제로 최근 연구에 따르면 환자들 스스로도 진료 결정 과정에 보다 적극적으로 참여하길 원하며[28], 의료진 역시 이러한 참여를 긍정적으로 수용하는 방향으로 변화하고 있다[23].

특히 임부는 자신의 건강뿐만 아니라 태아의 안녕을 함께 고려해야 하는 복합적인 책임을 지니고 있으며, 임신과 출산 과정에서 다양한 의료적 결정을 내려야 한다[29]. 현대 사회에서 임부는 인터넷과 모바일 기기를 활용하여 건강정보를 능동적으로 탐색하고 있으며, 이로 인해 의료 의사결정에 적극적으로 참여하려는 경향이 커지고 있다[30]. 이러한 상황에서 임부가 의료진과 협력하여 적절한 진료방향을 설정할 수 있도록 돕는 공유의사결정은 임부의 자기결정권을 존중하고 산과적 결과를 향상시키는 중요한 전략이다.

공유의사결정의 중요성은 오랫동안 논의되어 왔지만, 지금까지의 연구는 주로 의료진을 중심으로 이루어졌으며[31,32], 특히 특수한 생애주기 상황에 있는 임부를 대상으로 한 연구는 상대적으로 부족한 실정이다. 또한 팬데믹 이후 임부의 인터넷 활용률이 증가하는 가운데, 국내 임부의 e헬스 문해력 수준, 인터넷 건강정보에 대한 태도, 그리고 이러한 요소들이 공유의사결정에 미치는 영향에 대한 연구는 매우 제한적이다.

이에 본 연구는 임부를 대상으로 e헬스 문해력 수준과 인터넷 건강정보에 대한 태도를 파악하고 이러한 요소들이 공유의사결정에 어떠한 영향을 미치는지 규명하고자 한다. 또한 임부대상의 효과적인 건강정보 제공 전략 수립 및 건강증진 촉진을 위한 간호중재 개발에 기초자료를 제공하고자 한다.

본 연구의 구체적인 목적은 다음과 같다.

첫째, 대상자의 e헬스 문해력, 인터넷 건강정보에 대한 태도, 공유의사결정 정도를 파악한다.

둘째, 대상자의 특성에 따른 e헬스 문해력, 인터넷 건강정보에 대한 태도, 공유의사결정의 차이를 분석한다.

셋째, e헬스 문해력, 인터넷 건강정보에 대한 태도, 공유의사결정 간의 상관관계를 분석한다.

넷째, e헬스 문해력, 인터넷 건강정보에 대한 태도가 공유의사결정에 미치는 영향을 파악한다.

## Methods

**Ethics statement:** This study was approved by the Institutional Review Board of Ewha Womans University Mokdong Hospital (No. eumc-2024-08-008-003). Participation in the survey was considered as voluntary consent to the study.

### 연구 설계

본 연구는 임부를 대상으로 일반적 특성, 산과적 특성, e헬스 문해력, 인터넷 건강정보에 대한 태도, 공유의사결정을 확인하고, 이러한 요인들이 공유의사결정에 미치는 영향을 분석하기 위한 단면 연구이다. 연구의 기술은 STROBE 보고지침(https://www.strobe-statement.org/)에 따라 작성하였다.

### 연구 대상자

본 연구의 대상자는 서울시에 소재하는 상급 종합병원인 이화여자대학교 목동병원 산부인과를 이용하는 임산부를 대상으로 편의표집 하였다. 선정기준은 만 19세 이상의 성인 여성 중 서울시에 있는 이화여자대학교 목동병원 산부인과를 이용하는 임부이며, 한글을 읽고 응답할 수 없는 임부는 연구 대상에서 제외하였다.

본 연구에서 필요한 표본의 수는 G-power 3.1.9.7 프로그램을 이용하여 산출하였다. 다중선형 회귀분석에 필요한 최소 표본 수를 선행연구[33]를 참조하여 중간 정도의 효과크기 .15, 유의수준 .05, 검정력 .80, 예측 영향 변인(연령, 교육 수준, 결혼 여부, 임신 기간, 유산 경험 유뮤, 조산 경험 유무, 임신 방법, 고위험 임신 진단 여부, e헬스 문해력, 인터넷 건강정보에 대한 태도) 10개로 하였을 때 총 118명이 산출되었다. 참여자 수는 임부를 대상으로 온라인 설문조사를 시행한 선행연구에 근거하여[8] 탈락률 10%를 고려해 총 130명을 선정하였으며, 탈락 자료 없이 총 130명의 자료를 분석하였다.

### 연구 도구

본 연구의 도구는 도구의 개발자와 번안자에게 이메일로 도구 사용 승인을 받은 후 사용하였다.

### 공유의사결정

공유의사결정은 Suh와 Lee [33]가 개발한 도구를 사용하였다. 본 도구는 총 8문항으로 구성되어 있으며, 각 문항은 ‘전혀 그렇지 않은 편이다’ 1점부터 ‘매우 그런 편이다’ 5점까지의 Likert 척도로 응답한다. 점수의 범위는 8–40점으로, 각 항목의 점수를 합산한 총점이 높을수록 임부의 공유의사결정 수준이 높고 의료진과 환자 간의 의사소통이 잘 이루어지고 있음을 의미한다. 본 연구에서는 각 항목의 점수를 합산한 총점과 평균 점수를 확인하였다. 도구 개발 당시 신뢰도는 Cronbach’s α=.70이었고[33], 본 연구에서는 Cronbach’s α=.79였다.

### e헬스 문해력

e헬스 문해력은 Norman과 Skinner [13]의 도구를 Chang 등[34]이 한국어로 번역한 ‘한국어판 e헬스 문해력 척도(The eHealth Literacy Scale into Korean)’를 사용하였다. 본 도구는 총 8개의 문항으로 구성되어 있으며, 각 문항은 ‘전혀 아니다’ 1점부터 ‘매우 그렇다’의 5점으로 Likert 척도로 응답한다. 점수의 범위는 8–40점으로 각 항목의 점수를 합산한 총점이 높을수록 e-헬스 문해력이 높은 것을 의미하며, 본 연구에서는 각 항목의 점수를 합산한 총점과 평균 점수를 확인하였다. Norman과 Skinner [13]의 도구 개발 당시 신뢰도는 Cronbach’s α=.88이었으며[13], Chang 등[34]의 연구에서는 Cronbach’s α=.89, 본 연구에서는 Cronbach’s α=.85였다.

### 인터넷 건강정보에 대한 태도

인터넷 건강정보에 대한 태도는 Jung 등[35]이 개발한 도구를 사용하였다. 본 도구는 총 5문항으로 구성되어 있으며, 각 문항은 ‘전혀 아니다’ 1점부터 ‘매우 그렇다’ 5점까지의 Likert 척도로 응답한다. 이 도구는 인터넷 건강정보에 대한 개인의 생각과 태도를 측정하는 것이며, 점수의 범위는 5–25점으로 각 항목의 점수를 합산한 총점이 높을수록 인터넷 건강정보에 대해 긍정적이고 신뢰하는 태도가 높음을 의미한다. 본 연구에서는 각 항목의 점수를 합산한 총점과 평균 점수를 확인하였다. 개발 당시의 신뢰도는 제시되지 않았으나 Cronbach’s α=.70 이상임을 명시하였고[35], 이 도구를 사용한 Chang과 Im [36]의 연구에서는 Cronbach’s α=.80, 본 연구에서는 Cronbach’s α=.72였다.

### 대상자 특성

대상자 특성은 일반적 특성(연령, 교육 정도, 직업, 가족 구조, 결혼 상태 등) 7문항, 산과적 특성(임신 기간, 출산 횟수, 유산 경험, 고위험 임신 진단 등) 8문항으로 구성하였다.

### 자료 수집

본 연구의 자료 수집 기간은 2024년 10월 8일부터 10월 31일까지였으며, 연구 참여자 모집은 이화여자대학교 목동병원 산부인과 외래 및 병동 책임자에게 자료 수집 전 협조 요청을 받은 뒤 산부인과 외래와 병동 게시판에 모집 공고문을 게시하여 이루어졌다. 모집 공고문을 보고 대상자가 자발적으로 연구 참여 의사를 밝히면 연구 담당자가 연구 참여 설명문을 서면으로 제공하여 설명한 후, 대상자 본인이 직접 서면 동의서에 서명함으로써 연구 동의를 획득하였다. 서면 동의서 작성을 완료한 대상자에게 온라인 설문지를 URL 또는 QR 코드를 제공하여 참여할 수 있도록 하였다. 설문지 작성은 대상자가 사용하는 기기의 IP 주소를 이용하여 1인당 1회로 제한하였고, 설문을 완료한 대상자에게는 5,000원 상당의 소정의 모바일 상품권을 제공하였다.

### 자료 분석

본 연구에서 수집된 자료는 IBM SPSS 프로그램 ver. 29.0 (IBM Corp., Armonk, NY, USA)을 사용하여 분석하였다. 대상자의 일반적 특성, 산과적 특성, e헬스 문해력, 인터넷 건강정보에 대한 태도, 공유의사결정은 빈도와 백분율, 평균과 표준편차로 분석하였고, 대상자의 일반적 특성, 산과적 특성에 따른 e헬스 문해력, 인터넷 건강정보에 대한 태도, 공유의사결정의 차이는 독립 t 검정 및 일원 분산분석으로 분석하고, 사후 검정은 Scheffé test를 시행하였다. 대상자의 e헬스 문해력, 인터넷 건강정보에 대한 태도, 공유의사결정 간의 상관관계는 피어슨 상관계수(Pearson correlation coefficient)를 이용하여 분석하였고, 대상자의 특성과 e헬스 문해력, 인터넷 건강정보에 대한 태도가 공유의사결정에 미치는 영향을 확인하기 위해 모델을 나누어 위계적 다중선형회귀(hierarchical multiple linear regression) 분석을 시행하였다.

## Results

### 대상자의 특성

대상자의 평균 연령은 31.73±2.86세였으며, 연령대로 분류하면 30세 이상 35세 미만이 78명(60.0%)으로 가장 많았다. 최종 학력은 고등학교 졸업이 24명(18.5%), 대학교 졸업이 100명(76.9%), 대학원 졸업 이상이 6명(4.6%)으로 나타났다. 종교는 무교 67명(51.5%), 직업은 있음이 86명(66.2%)이었으며, 결혼 상태는 기혼 127명(97.7%), 미혼 3명(2.3%)으로 나타났다. 가족 구조는 핵가족이 122명(93.8%), 월수입은 300만 원 이상 400만 원 미만이 48명(36.9%)으로 가장 많았다.

대상자의 산과적 특성에서 현재 임신 기간은 임신 초기(5–13주)가 39명(30.0%), 임신 중기(14–28주)가 49명(37.7%), 임신 후기(29–40주)가 42명(32.3%)으로 비교적 고르게 나타났다. 출산 횟수는 출산 경험이 없는 경우가 114명(87.7%)이었고, 대부분 유산 경험이나 조산 경험이 없었다(각각 113명, 86.9%; 125명, 96.2%). 임신은 107명(82.3%)이 계획된 임신이었으며, 114명(87.7%)이 자연 임신이었다. 대상자중 11명(8.5%)이 고위험 임신 진단을 받았다(Table 1).

### 대상자의 특성에 따른 e헬스 문해력, 인터넷 건강정보에 대한 태도, 공유의사결정

e헬스 문해력 점수의 평균은 총점 40점 만점에 32.96±4.22, 각 항목당 5점 척도의 기준으로 한 총점의 평균은 4.11±0.75로 높은 수준으로 나타났다. 각 질문의 항목 중 ‘나는 건강과 관련된 궁금증에 대한 답을 찾기 위해 인터넷을 사용하는 방법을 알고 있다.’라는 문항이 평균 4.26±0.72로 가장 높았다. 인터넷 건강정보에 대한 태도 점수의 평균은 총점 25점 만점에 20.53±2.54, 각 항목당 5점 척도의 기준으로 한 총점의 평균은 4.10±0.73로 높은 수준으로 나타났다. 각 질문의 항목 중 ‘인터넷에서 찾았던 건강정보는 실제로 도움이 된다.’ 문항이 평균 4.19±0.71로 가장 높았다. 공유의사결정에 대한 점수의 평균은 점수의 평균은 총점 40점 만점에 33.38±3.75, 각 항목당 5점 척도로 측정한 총점의 평균은 4.17±0.73로 높은 수준이었으며, 이 중 ‘의사는 치료법과 관련 부작용에 대해 충분히 설명했다.’라는 문항이 평균 4.31±0.73으로 가장 높았다(Table 2).

e헬스 문해력은 대상자의 특성 중 임신 방법(F=4.30, *p*=.040)과 고위험 임신 진단(F=6.97, *p*=.009)에서 유의한 차이가 있었다. 자연 임신한 대상자들이 난임 시술한 대상자들보다 평균 점수가 유의하게 높았으며, 고위험 임신 진단을 받지 않은 대상자들이 진단받은 대상자들보다 점수가 유의하게 높았다(Table 1).

인터넷 건강정보에 대한 태도는 대상자의 가족 구조(F=4.99, *p*=.008)에 따라 유의한 차이를 보였으며, 대가족인 대상자들이 핵가족이나 혼자 거주하는 대상자들보다 평균 점수가 유의하게 높은 것으로 나타났다. 현재 임신 기간이 임신 초기인 대상자들이 평균 점수가 제일 높았으며(F=7.30, *p*<.001), 고위험 진단을 받지 않은 대상자들이 진단받은 대상자들보다 유의하게 점수가 높았다(F=6.99, *p*=.009) (Table 1).

공유의사결정은 대상자의 연령(F=3.14, *p*=.047)과 결혼 상태(F=4.31, *p*=.040)에 따라 통계적으로 유의한 차이를 보였다. 연령이 높은 경우 공유의사결정의 평균 점수가 유의하게 높았으며, 기혼자가 미혼자보다 평균 점수가 유의하게 높은 것으로 나타났다(Table 1).

### e헬스 문해력, 인터넷 건강정보에 대한 태도, 공유의사결정 간의 상관관계

대상자의 e헬스 문해력과 공유의사결정은 유의한 양의 상관관계가 나타났으며(r=.67, *p*<.001), 인터넷 건강정보에 대한 태도 또한 공유의사결정과 유의한 양의 상관관계를 보였다(r=.71, *p*<.001). 이는 e헬스 문해력과 인터넷 건강정보에 대한 태도가 높을수록 공유의사결정 정도도 높다는 것을 의미한다. 대상자의 e헬스 문해력과 인터넷 건강정보에 대한 태도 간에도 유의한 양의 상관관계를 보여(r=.80, *p*<.001) e헬스 문해력이 높은 경우 인터넷 건강정보에 대한 태도도 긍정적인 것으로 나타났다(Table 3).

### 공유의사결정에 영향을 미치는 요인

공유의사결정에 영향을 미치는 요인을 확인하기 위해 모델 Ⅰ은 일반적 특성에서 유의한 차이를 보였던 연령을 투입하였고, 일반적 특성 중에서 월수입, 직업 유무, 교육 정도 변수를, 산과적 특성 중에서 분만 경험, 유산 경험, 조산 경험, 임신 형태, 고위험 임신 진단 유무 변수를 더미 변수 처리하여 통제변수로 포함하였다. 결혼 여부 변수는 응답자 중 미혼인 경우가 3명으로 적어 포함하지 않았다. 이후 모델 Ⅱ에는 e헬스 문해력, 인터넷 건강정보에 대한 태도를 추가하여 위계적 다중선형회귀분석을 실시하였다. 모델 Ⅰ 회귀식은 유의하였고(F=4.04 *p*<.001) 변수들의 설명력은 20.7%였는데 변수 중 연령(β=.20, *p*=.029) 만이 공유의사결정에 영향을 미치는 요인으로 나타났다. e헬스 문해력과 인터넷 건강정보에 대한 태도를 추가한 모델 Ⅱ에서는 고위험 임신 진단(유) (β=.19, *p*=.033), e헬스 문해력(β=.27, *p*=.015), 인터넷 건강정보에 대한 태도(β=.39, *p*<.001)가 공유의사결정에 영향을 미치는 요인으로 나타났다. 모델 Ⅱ 회귀식은 유의하였고(F=41.03, *p*<.001), 설명력은 46.3%였다(Table 4).

## Discussion

본 연구는 임부의 e헬스 문해력, 인터넷 건강정보에 대한 태도를 파악하고, 이러한 요인들이 공유의사결정에 미치는 영향을 분석하기 위해 시행하였다.

임부의 공유의사결정에 미치는 영향을 알아보기 위해 위계적 다중선형회귀분석을 실시한 결과 고위험 임신 진단이 있는 경우 공유의사결정이 높게 나타났다. 산과적 특성에 따른 공유의사결정에 관한 연구는 미비하여 직접적인 비교는 어렵지만, 고위험 임신 진단으로 인한 본인과 태아의 건강에 관한 불확실성, 불안을 극복하기 위해 임부가 의료진과의 의사결정에 적극적으로 참여한다는 선행연구[37]와 유사한 결과이다.

또한, e헬스 문해력이 높고 인터넷 건강정보에 대해 긍정적인 태도를 가진 임부일수록 공유의사결정 정도가 높은 것으로 나타났다. 이는 건강정보를 효과적으로 수집·해석·활용할 수 있는 능력이 공유의사결정 참여에 중요한 역할을 한다는 것을 의미한다. 특히 인터넷 건강정보에 대해 긍정적인 태도는 의료진과의 소통을 보다 능동적으로 이끄는 요인으로 작용할 수 있다[38].

본 연구에서 임부의 공유의사결정 정도를 조사한 결과, 총점 40점 만점에 33.38±3.75, 5점 만점에 평균 4.17점으로 나타났는데, 이는 성인 남녀를 대상으로 한 선행연구[39]의 3.47, 암 환자를 대상으로 한 연구[40]의 3.39, 당뇨병 환자를 대상으로 한 연구[23] 3.01보다 높았다. 본 연구에서 연령이 높을수록, 미혼보다 기혼이 통계적으로 유의하게 높게 나타났는데 이는 기혼자의 경우 태아와 가족의 건강에 대한 책임감이 공유의사결정 참여를 높이는 요인이 될 수 있음을 시사한다. 이 결과는 연령이 낮을수록 공유의사결정이 높다고 보고한 연구[39]와는 상반된 결과이다. Devoe 등[41]은 연령이 높을수록 의료진에게 설명을 요구하는 소통을 하고, 연령이 낮으면 의료진과의 자발적 소통을 많이 한다는 연구 결과를 보고하였다. 우리나라 임부의 평균 연령이 점차 높아지고 있는 점을 고려할 때, 연령에 따른 공유의사결정의 차이에 대한 후속 연구가 필요할 것으로 보인다.

임부의 e헬스 문해력은 총점 40점 만점에 32.96±4.22, 5점 만점에 평균 4.11점으로 나타났는데, 이는 국외의 임부를 대상으로 한 연구 결과[15,38]보다 높은 수준이다. 하위 문항 중 ‘나는 건강과 관련된 궁금증에 대한 답을 찾기 위해 인터넷 사용 방법을 알고 있다.’라는 항목이 4.26점으로 가장 높았고, ‘나는 인터넷에서 어떠한 건강 관련 자원이 이용 가능한지 알고 있다.’라는 항목이 3.95점으로 가장 낮았다. 이는 임부들이 정보 검색 능력은 높지만, 그 정보를 선별하고 해석하여 개인의 건강 상황에 적용하는 데에는 어려움을 겪고 있음을 나타낸다. 실제 국내 연구[7]에서도 이와 유사하게 정보 해석 및 활용 능력에서 낮은 점수를 보였다. 선행연구에서 e헬스 문해력은 연령과 교육 수준에 따라 높아질 수 있다고 제시하였으나[15,42], 본 연구에서는 유의한 차이가 나타나지 않았다. 다만 임신 방법과 고위험 임신 여부에서는 유의한 차이를 보여, 자연 임신군보다 난임군, 고위험 임신군에서 e헬스 문해력이 낮게 나타났다. 이는 건강 취약 집단일수록 부정확하거나 신뢰할 수 없는 온라인 건강정보에 더 취약하며[43], 이로 인해 정보의 해석 및 활용에 어려움을 겪을 수 있음을 시사한다[44,45]. 따라서 임부와 같은 취약 계층, 더불어 난임이나 고위험 임신을 진단받은 상황의 임부들에게는 정확하고 신뢰성 있는 정보를 제공하는 것이 e헬스 문해력을 높이는 것으로 여겨진다.

인터넷 건강정보에 대한 태도는 총점 25점 만점에 20.53±2.54, 5점 만점에 평균 4.10점으로 일반 직장인을 대상으로 연구[12]인 평균 3.09점보다 높았다. 직장인과 직접적인 비교를 하긴 어렵지만, 이는 신체적 변화와 건강에 대한 관심이 높은 임부들이 직장인보다 인터넷을 통한 건강정보 탐색에 더 적극적이기 때문으로 해석된다. 그러나 ‘인터넷에서 찾았던 건강정보는 신뢰할 만하다‘의 문항에서 가장 낮은 점수(4.06점)가 나타났으며, 선행연구[12]에서도 해당 문항이 가장 낮은 점수(2.91점)를 기록하였다. 이는 임부들이 인터넷 건강정보를 적극적으로 활용하고 있음에도, 정보의 신뢰성에 대해서는 여전히 의문을 가지고 있음을 보여준다.

또한, 현재 임신 주수가 낮고 고위험 임신 진단을 받지 않은 경우 인터넷 건강정보에 대한 태도가 유의하게 긍정적으로 나타났다. 이는 임신 초기일수록 정보 탐색의 양이 적고, 인터넷에서 얻은 정보를 상대적으로 신뢰하는 경향이 반영된 것으로 보인다. 정보의 과잉은 오히려 혼란을 초래하고 정보에 대한 불신을 유발할 수 있으며[46], 이것을 고위험 임신군에서 인터넷 건강정보에 대한 태도가 낮은 이유로 해석할 수 있다. 본 연구에서도 e헬스 문해력과 인터넷 건강정보에 대한 태도 간에는 유의한 정적 상관관계가 있었으며, 이는 e헬스 문해력이 높을수록 인터넷 건강정보에 대한 신뢰와 긍정적 태도가 향상된다는 선행연구[47]의 결과와 일치한다.

이러한 결과는 임부의 e헬스 문해력 향상이 건강정보 활용 능력 및 인터넷 건강정보에 대한 태도 개선에 중요한 기초가 되며, 이는 궁극적으로 공유의사결정 향상으로 이어질 수 있음을 시사한다. 임부, 특히 고위험 임신군이나 난임 여성과 같은 취약 집단에게는 정확하고 신뢰할 수 있는 건강정보를 제공하고, e헬스 문해력 향상 교육을 강화할 필요가 있다. 따라서 임부들을 위한 건강정보 검색 및 활용 교육 프로그램 개발이 맞춤형 교육과 정확한 정보를 수집하는 데 도움을 줄 것으로 보인다. 더불어 임상에서는 임부 전문 상담사를 양성하고 배치한다면 잘못된 정보로부터의 혼동을 예방하고 올바른 결정을 통해 건강한 임신과 출산을 도모할 수 있을 것이다.

본 연구는 이러한 필요성을 인식하고 임부를 대상으로 e헬스 문해력과 인터넷 건강정보에 대한 태도를 조사하여 기초 자료료 제시한다는 의의가 있지만, 수도권 지역에 있는 특정 상급 종합병원 산부인과를 방문하는 임부들만을 대상으로 진행되어 연구 결과를 일반화하기는 어렵다. 또한 임신 분기와 상관없이 고위험 임부와 정상 임부를 모두 포함하여 진행하였기 때문에, 각각의 특성을 고려한 추가 연구가 필요하다.

## Figures and Tables

**Table 1.** Differences in eHealth literacy, attitudes toward internet health information, and shared decision-making according to participants’ characteristics (N=130)

Characteristic	Categories	n (%)	eHealth literacy		Attitudes toward internet health information		Shared decision-making	
			Mean±SD	t/F (*p*)	M±SD	t/F (*p*)	Mean±SD	t/F (*p*)
Age (year)	<30^a^	27 (20.8)	3.95±0.61	1.75 (.178)	3.96±0.60	1.40 (.250)	3.98±0.60	3.14 (.047) (a<b)^†^
	≥30, <35^b^	78 (60.0)	4.17±0.52		4.13±0.48		4.21±0.43	
	≥35^c^	25 (19.2)	4.14±0.43		4.16±0.44		4.25±0.35	
Education level	High school graduation	24 (18.5)	4.19±0.67	0.55 (.579)	4.26±0.54	1.69 (.188)	4.21±0.61	0.22 (.807)
	University graduation	100 (76.9)	4.09±0.49		4.06±0.49		4.15±0.43	
	≥Master’s	6 (4.6)	4.27±0.37		4.20±0.43		4.25±0.44	
Religion	None	67 (51.5)	4.01±0.51	6.12 (.055)	4.04±0.49	2.03 (.156)	4.09±0.49	3.91 (.050)
	Yes	63 (48.5)	4.23±0.52		4.17±0.51		4.25±0.47	
Job	Yes	86 (66.2)	4.12±0.49	0.04 (.085)	4.10±0.48	0.02 (.905)	4.16±0.43	0.16 (.691)
	No	44 (33.8)	4.10±0.60		4.11±0.55		4.19±0.55	
Marital status	Married	127 (97.7)	4.13±0.51	3.23 (.075)	4.11±0.50	1.66 (.200)	4.18±0.45	4.31 (.040)
	Unmarried	3 (2.3)	3.58±0.94		3.73±0.64		3.63±0.87	
Family structure	Nuclear family	122 (93.8)	4.12±0.53	1.82 (.167)	4.10±0.49	4.99 (.008)	4.17±0.47	1.77 (.179)
	Extended family	7 (5.4)	4.14±0.38		4.25±0.41		4.30±0.33	
	Alone	1 (0.8)	3.12		2.6		3.37	
Monthly household income (million KRW)	<2.9	42 (32.3)	4.03±0.57	1.08 (.342)	4.07±0.56	0.45 (.637)	4.21±0.51	2.25 (.109)
	≥3, <4.9	57 (43.9)	4.13±0.46		4.09±0.50		4.08±0.41	
	≥5	31 (23.8)	4.21±0.58		4.18±0.83		4.29±0.48	
Current pregnancy period (weeks)	5–13^a^	39 (30.0)	4.21±0.38	2.08 (.130)	4.29±0.37	7.30 (<.001) (a>c)^†^	4.26±0.27	1.56 (.215)
	14–28^b^	49 (37.7)	4.16±0.53		4.13±0.47		4.17±0.51	
	29–40^c^	42 (32.3)	3.99±0.62		3.89±0.58		4.08±0.54	
Parity	Nullipara	114 (87.7)	4.12±0.53	0.01 (.931)	4.09±0.50	0.62 (.432)	4.16±0.46	0.39 (.531)
	Multipara	16 (22.3)	4.11±0.46		4.20±0.53		4.24±0.48	
Abortion history	No	113 (86.9)	4.13±0.52	0.67 (.413)	4.12±0.50	1.23 (.261)	4.16±0.48	0.43 (.513)
	Yes	17 (13.1)	4.02±0.58		3.97±0.55		4.24±0.36	
Premature delivery history	No	125 (96.2)	4.11±0.54	0.20 (.653)	4.10±0.52	0.01 (.951)	4.16±0.47	1.51 (.221)
	Yes	5 (3.8)	4.22±0.56		4.12±0.10		4.25±0.20	
Planned pregnancy	No	23 (17.7)	4.09±0.63	0.08 (.782)	4.03±0.54	0.55 (.460)	4.14±0.57	0.13 (.721)
	Yes	107 (82.3)	4.13±0.50		4.12±0.50		4.17±0.44	
Pregnancy method	Natural	114 (87.7)	4.16±0.51	4.30 (.040)	4.12±0.49	2.03 (.157)	4.18±0.45	1.02 (.315)
	Infertility treatment	16 (12.3)	3.87±0.60		3.93±0.58		4.06±0.54	
High risk pregnancy	No	119 (91.5)	4.16±0.48	6.97 (.009)	4.14±0.48	6.99 (.009)	4.17±0.43	0.01 (.918)
	Yes	11 (8.5)	3.73±0.82		3.72±0.58		4.16±0.74	

KRW: Korean won (1 million KRW is approximately 731 US dollars).

† Scheffé test.

**Table 2.** Scores of participants’ eHealth literacy, attitudes toward internet health information, and shared decision-making (N=130)

Categories	Range	Mean±SD
eHealth literacy	5–40	32.96±4.22
	1–5	4.11±0.75
I know how to use the internet to find answers to my health–related questions.	1–5	4.26±0.72
I know what kinds of health–related resources are available on the internet	1–5	3.95±0.69
Attitudes toward internet health information	5–25	20.53±2.54
	1–5	4.10±0.73
The health information I found on the internet was actually helpful.	1–5	4.19±0.71
The health information I found on the internet was trustworthy.	1–5	4.06±0.70
Shared decision-making	5–40	33.38±3.75
	1–5	4.17±0.73
The doctor adequately explained the treatment methods and their potential side effects.	1–5	4.31±0.73
I fully expressed my opinion to the doctor regarding the possible treatment alternatives.	1–5	4.08±0.77

**Table 3.** Relationships among shared decision-making, eHealth literacy, and attitudes toward internet health information (N=130)

Variable	Shared decision-making	eHealth literacy
	r (*p*)	r (*p*)
Shared decision-making	1	
eHealth literacy	.67 (<.001)	1
Attitudes toward internet health information	.71 (<.001)	.80 (<.001)

**Table 4.** Factors influencing shared decision-making (N=130)

Variable	Model Ⅰ					Model Ⅱ				
	B	SE	β	t	*p*	B	SE	β	t	*p*
Age	.19	.02	.20	1.46	.029	.01	.01	.03	0.43	.670
Monthly household income ≥3 million KRW (reference, <3 million KRW)	–.12	.11	–.11	–1.07	.285	–.14	.07	–.14	–1.89	.062
Job (reference, none)	.12	.09	.12	1.40	.165	.06	.07	.06	0.87	.387
≥University graduation (reference, <high school graduation)	.00	.10	.01	0.03	.974	.05	.08	.04	0.69	.494
Multipara (reference, nullipara)	.19	.13	.12	1.54	.182	.08	.08	.05	0.81	.524
Abortion history (reference, no)	–.06	.11	–.04	–0.51	.613	.11	.09	.08	1.19	.237
Premature delivery history (reference, no)	.32	.20	.13	1.63	.106	.24	.15	.10	1.58	.118
Infertility treatment (reference, natural)	–.13	.14	–.09	–0.96	.337	–.03	.11	–.02	–0.26	.799
High risk pregnancy (reference, no)	.07	.15	.04	0.46	.644	.20	.10	.19	2.36	.033
eHealth literacy						.24	.10	.27	2.48	.015
Attitudes toward internet health information						.31	.09	.39	3.92	<.001
R^2^ (adjusted R^2^)	.35 (.207)					.52 (.463)				
F (*p*)	4.04 (<.001)					41.03 (<.001)				

Model Ⅰ: general and obstetric characteristics. Model Ⅱ : eHealth literacy and attitudes toward internet health information are added. KRW: Korean won (1 million KRW is approximately 731 US dollars).
